# Mortality after transcatheter versus surgical aortic valve replacement: an updated meta-analysis of randomised trials

**DOI:** 10.1007/s12471-020-01378-1

**Published:** 2020-03-12

**Authors:** H. Takagi, Y. Hari, K. Nakashima, T. Kuno, T. Ando, ALICE (All-Literature Investigation of Cardiovascular Evidence) Group

**Affiliations:** 1 Department of Cardiovascular Surgery, Shizuoka Medical Center, Shizuoka, Japan; 2 Department of Cardiovascular Surgery, Kitasato University School of Medicine, Sagamihara, Japan; 3 Department of Medicine, Mount Sinai Beth Israel Medical Center, New York, NY USA; 4 Division of Interventional Cardiology, Department of Cardiology, New York Presbyterian Hospital/Columbia University Medical Center, New York, NY USA

**Keywords:** Meta-analysis, Randomised controlled trial, Surgical aortic valve replacement, Transcatheter aortic valve implantation

## Abstract

**Background:**

To determine whether transcatheter aortic valve implantation (TAVI) improves early (30-day) and midterm (1-year) mortality compared with surgical aortic valve replacement (SAVR), we performed an updated meta-analysis of all the currently available randomised controlled trials (RCTs).

**Methods:**

To identify all RCTs providing both 30-day and 1‑year mortality after TAVI versus SAVR, PubMed and ClinicalTrials.gov were searched up to and including July 2019. A risk difference (RD) and its 95% confidence interval were generated using data of prespecified outcomes in both the TAVI and SAVR groups. Study-specific estimates were pooled using inverse variance-weighted averages of RDs in the random-effects model.

**Results:**

We identified seven eligible high-quality RCTs including a total of 7631 as-treated patients. Pooled analyses demonstrated significantly lower 30-day (RD −0.60%; *p* = 0.046) and 1‑year all-cause mortality (RD −1.12%; *p* = 0.03) after TAVI than after SAVR. No funnel plot asymmetry was detected for 30-day and 1‑year mortality. Meta-regression analyses indicated that RDs of 30-day and 1‑year mortality between TAVI and SAVR were not modulated by mean Society of Thoracic Surgeons Predicted Risk of Mortality score. Bleeding complications at 30 days and 1 year and stage 2/3 acute kidney injury at 30 days were significantly less frequent after TAVI than after SAVR, whereas major vascular complications and new permanent pacemaker implantation at 30 days and 1 year were significantly more frequent after TAVI than after SAVR.

**Conclusion:**

The best evidence from the present meta-analysis of all the currently available RCTs suggests that TAVI may reduce 30-day and 1‑year all-cause mortality compared with SAVR.

**Electronic supplementary material:**

The online version of this article (10.1007/s12471-020-01378-1) contains supplementary material, which is available to authorized users.

## What’s new?

- To determine whether transcatheter aortic valve implantation (TAVI) improves early (30-day) and midterm (1-year) mortality compared with surgical aortic valve replacement (SAVR), we performed an updated meta-analysis of all the currently available randomised controlled trials (RCTs).
- We identified seven eligible high-quality RCTs including a total of 7631 as-treated patients.
- None of the included RCTs showed significantly lower all-cause mortality after TAVI than after SAVR.
- Pooled analyses demonstrated significantly lower 30-day [risk difference (RD) −0.60%; *p* = 0.046] and 1‑year all-cause mortality (RD −1.12%; *p* = 0.03) after TAVI than after SAVR.

## Introduction

Because it is a less invasive procedure, transcatheter aortic valve implantation (TAVI) was introduced as a substitute for surgical aortic valve replacement (SAVR) in high surgical risk patients with severe aortic stenosis (AS) and was expected to achieve at least equivalent or if possible better postprocedural prognosis. As far as we know, however, neither randomised controlled trials (RCTs) of TAVI versus SAVR nor meta-analyses [1–4] of RCTs have reported significantly lower mortality after TAVI than after SAVR to date. Recently (in 2019), two novel RCTs, the Evolut Low Risk trial [5] and the Placement of Aortic Transcatheter Valves (PARTNER) 3 trial [6], provided outcomes after TAVI versus SAVR. In the present article, to determine whether TAVI improves early (30-day) and midterm (1-year) mortality compared with SAVR, we performed an updated meta-analysis of all the currently available RCTs including the two above-mentioned recently reported RCTs [5, 6].

## Methods

To identify all RCTs providing both 30-day and 1‑year mortality after TAVI versus SAVR for AS patients, PubMed (https://www.ncbi.nlm.nih.gov/pubmed/) and ClinicalTrials.gov (https://clinicaltrials.gov/ct2/home) were searched up to and including July 2019. Search terms included ‘transcatheter’, ‘aortic valve’ ‘implantation(s) or replacement(s)’ and ‘randomised’. Studies meeting the following criteria were included in a meta-analysis: the design was an RCT; the study population consisted of AS patients; patients were randomised to TAVI versus SAVR; outcomes included both 30-day and 1‑year all-cause mortality. A risk difference (RD) and its 95% confidence interval (CI) were generated using data of prespecified outcomes in both the TAVI and SAVR groups. Study-specific estimates were pooled using inverse variance-weighted averages of RDs in the random-effects model. In the present study, the primary end point was all-cause mortality, and the secondary end points included myocardial infarction, stroke, bleeding complications, acute kidney injury (AKI), vascular complications, and new permanent pacemaker implantation (PMI). When the number of studies reporting an end point was <3, we did not perform pooled analysis for the end point. Funnel plot asymmetry (suggesting publication bias) was mathematically examined using the linear regression test. To assess whether mean surgical risk [Society of Thoracic Surgeons Predicted Risk of Mortality (STS-PROM) score] of patients and proportion of patients undergoing trans(ilio)femoral TAVI (TF-TAVI) modulate study-specific estimates (RDs of mortality between TAVI and SAVR), a random-effects restricted-maximum likelihood meta-regression analysis was conducted. All analyses were performed using Review Manager version 5.3 (available from http://tech.cochrane.org/revman) and Comprehensive Meta-Analysis version 3 (Biostat, Englewood, NJ, USA).

## Results

The STACCATO trial (prospective, randomised trial of transapical transcatheter aortic valve implantation vs surgical aortic valve replacement in operable elderly patients with aortic stenosis) [7] was not registered in ClinicalTrials.gov. Furthermore, the study was unexpectedly terminated after including only 70 patients and did not report 1‑year outcomes. Thus, we decided to exclude this truncated RCT [7], and accordingly seven eligible high-quality RCTs ([5, 6, 8–12]; Tab. 1) were included in the present meta-analysis. Three RCTs (Evolut Low Risk [5], Nordic Aortic Valve Intervention (NOTION) [8], and PARTNER 3 [6]) consisted of patients at low surgical risk (STS-PROM <4%), three RCTs (PARTNER 2 [10], Surgical Replacement and Transcatheter Aortic Valve Implantation (SURTAVI) [11], and U.S. CoreValve [12]) were composed of those at intermediate surgical risk (STS-ROM 4–8%), and only one RCT (PARTNER 1 [9]) was made up of those at high surgical risk (STS-ROM ≥8%). The primary analysis in each RCT was conducted in the as-treated population in five studies [5, 6, 8, 11, 12] and in the intention-to-treat population in two studies [9, 10]. Hence, we determined to extract data in the as-treated population from all the seven RCTs including a total of 7631 patients. The principal analysis of the present study pooled data from the as-treated population, and the sensitivity analysis combined data from the intention-to-treat population. We performed another sensitivity analysis excluding the PARTNER 1 trial [9] (including patients at high surgical risk) from the principal analysis (as-treated population) of the primary end point (all-cause mortality). Details of the primary and secondary end points are listed in Tab. 1 and Table S1 (Electronic Supplementary Material). Results of the principal and sensitivity analysis are summarised in Tab. 2.

**Table 1:** Study design and primary end point (all-cause mortality)

Study	ClinicalTrials.gov number	Primary end point	Principal analysis population	As-treated population				Intention-to-treat population				TF-TAVI (%)	All-cause mortality															
				Number		STS-PROM (%)		Number		STS-PROM (%)			Principal analysis (as-treated population)								Sensitivity analysis (intention-to-treat population)							
				TAVI	SAVR	TAVI	SAVR	TAVI	SAVR	TAVI	SAVR		30 days				1 year				30 days				1 year			
													Number		Percentage		Number		Percentage		Number		Percentage		Number		Percentage	
													TAVI	SAVR	TAVI	SAVR	TAVI	SAVR	TAVI	SAVR	TAVI	SAVR	TAVI	SAVR	TAVI	SAVR	TAVI	SAVR
Evolut Low Risk 2019 [5]	NCT02701283	Composite of all-cause mortality or disabling stroke at 2 years	As-treated	725	678	1.9 ± 0.7	1.9 ± 0.7	734	734	1.9 ± 0.7	1.9 ± 0.7	99.0	4	9	0.6	1.3	17	20	2.3	2.9	4	6	0.5	0.8	18	21	2.5	2.9
NOTION 2015 [8]	NCT01057173	Composite of all-cause mortality, stroke, or MI at 1 year	As-treated	142	134	Unavailable		145	135	2.9 ± 1.6	3.1 ± 1.7	96.5	3	5	2.1	3.7	7	10	4.9	7.5	6	6	4.1	4.4	Unavailable		Unavailable	
PARTNER 1 2011 [9]	NCT00530894	All-cause mortality at 1 year	Intention-to-treat	344	313	Unavailable		348	351	11.8 ± 3.3	11.7 ± 3.5	70.1	18	25	5.2	8.0	81	78	23.5	24.9	12	22	3.4	6.3	84	89	24.1	25.4
PARTNER 2 2016 [10]	NCT01314313	Composite of all-cause mortality or disabling stroke at 2 years	Intention-to-treat	994	944	Unavailable		1011	1021	5.8 ± 2.1	5.8 ± 1.9	76.7	34	38	3.4	4.0	117	121	11.8	12.8	39	41	3.9	4.0	123	124	12.2	12.1
PARTNER 3 2019 [6]	NCT02675114	Composite of all-cause mortality, stroke, or rehospitalisation at 1 year	As-treated	496	454	1.9 ± 0.7	1.9 ± 0.6	503	497	Unavailable		100	2	5	0.4	1.1	5	11	1.0	2.4	2	5	0.4	1.0	Unavailable		Unavailable	
SURTAVI 2017 [11]	NCT01586910	Composite of all-cause mortality or disabling stroke at 2 years	As-treated	864^a^	796^a^	4.4 ± 1.5	4.5 ± 1.6	879	867	4.4 ± 1.5	4.5 ± 1.6	93.6	19	14	2.2	1.8	58	54	6.7	6.8	18	11	2.0	1.3	62	59	7.1	6.8
U.S. CoreValve 2014 [12]	NCT01240902	All-cause mortality at 1 year	As-treated	390	357	7.3 ± 3.0	7.5 ± 3.2	394	401	7.3 ± 3.0	7.5 ± 3.4	82.8	13	16	3.3	4.5	55	67	14.1	18.8	Unavailable		Unavailable		Unavailable		Unavailable	

*MI* myocardial infarction, *NOTION* Nordic Aortic Valve Intervention, *PARTNER* Placement of Aortic Transcatheter Valves, *SAVR* surgical aortic valve replacement, *STS-PROM* Society of Thoracic Surgeons Predicted Risk of Mortality, *SURTAVI* Surgical Replacement and Transcatheter Aortic Valve Implantation, *TAVI* transcatheter aortic valve implantation, *TF* trans(ilio)femoral

**Table 2:** Summary of the principal and sensitivity analysis of primary and secondary end points

End point				Principal analysis						Sensitivity analysis										
				As-treated population						Intention-to-treat population						As-treated population (excluding PARTNER 1 2011 [9])				
				Study (*n*)	RD (%)	LLCI (%)	ULCI (%)	*p* value	Figure	Study (*n*)	RD (%)	LLCI (%)	ULCI (%)	*p* value	Figure	Study (*n*)	RD (%)	LLCI (%)	ULCI (%)	*p* value
Primary	All-cause mortality	30 ays		7	−0.60	−1.20	−0.01	0.05 (0.046)^*^	1	6	−0.23	−0.85	0.40	0.48	S1	6	−0.55	−1.15	0.05	0.07
		1 year		7	−1.12	−2.12	−0.11	0.03^*^	1	4	−0.24	−1.51	1.04	0.72	S1	6	−1.11	−2.13	−0.09	0.03^*^
Secondary	MI	30 days		7	−0.34	−0.78	0.09	0.12	S2	4	−0.16	−0.64	0.32	0.52	S3					
		1 year		4	−0.04	−0.81	0.74	0.93	S2	4	−0.13	0.74	0.49	0.69	S3					
	Stroke	30 days		7	−0.56	−1.54	0.41	0.26	S4	4	−0.43	−2.14	1.28	0.62	S5					
		1 year		7	−0.72	−1.98	0.53	0.26	S4	4	0.15	−1.38	1.69	0.85	S5					
	BC	Major	30 days	3	−10.50	−13.18	−7.82	<0.00001^*^	S6	1	Not performed				–					
			1 year	3	−9.78	−14.42	−5.15	<0.0001^*^	S7	1	Not performed				–					
		LT or disabling	30 days	3	−18.35	−32.52	−4.18	0.01^*^	S6	1	Not performed				–					
			1 year	3	−16.40	−32.24	−0.56	0.04^*^	S7	1	Not performed				–					
		Major, LT, or disabling	30 days	3	−19.88	−28.45	−11.32	<0.00001^*^	S6	0	–				–					
			1 year	2	Not performed				–	0	–				–					
	AKI	Creatinine >3 mg/dl	30 days	1	Not performed				–	1	Not performed				–					
			1 year	1	Not performed				–	1	Not performed				–					
		Stage 3	30 days	2	Not performed				–	1	Not performed				–					
			1 year	1	Not performed				–	1	Not performed				–					
		Stage 2 or 3	30 days	3	−2.09	−3.61	−0.56	0.007^*^	S8	0	–				–					
			1 year	1	Not performed				–	0	–				–					
		Any	30 days	1	Not performed				–	0	–				–					
			1 year	1	Not performed				–	0	–				–					
	MVC	30 days		5	2.56	0.50	4.61	0.01^*^	S9	2	Not performed				–					
		1 year		4	2.48	0.19	4.77	0.03^*^	S9	2	Not performed				–					
	NPPMI	30 days		6	8.89	3.02	14.75	0.003^*^	S10	2	Not performed				–					
		1 year		6	9.25	2.74	15.77	0.005^*^	S10	2	Not performed				–					

When the number of studies reporting an end point was <3, we did not perform pooled analysis for the end point

*AKI* acute kidney injury, *BC* bleeding complications, *LLCI* lower limit of confidence interval, *LT* life-threatening, *MI* myocardial infarction, *MVC* major vascular complications, *NPPMI* new permanent pacemaker implantation, *RD* risk difference, *ULCI* lower limit of confidence interval

*Statistically significant

**Fig. 1:**
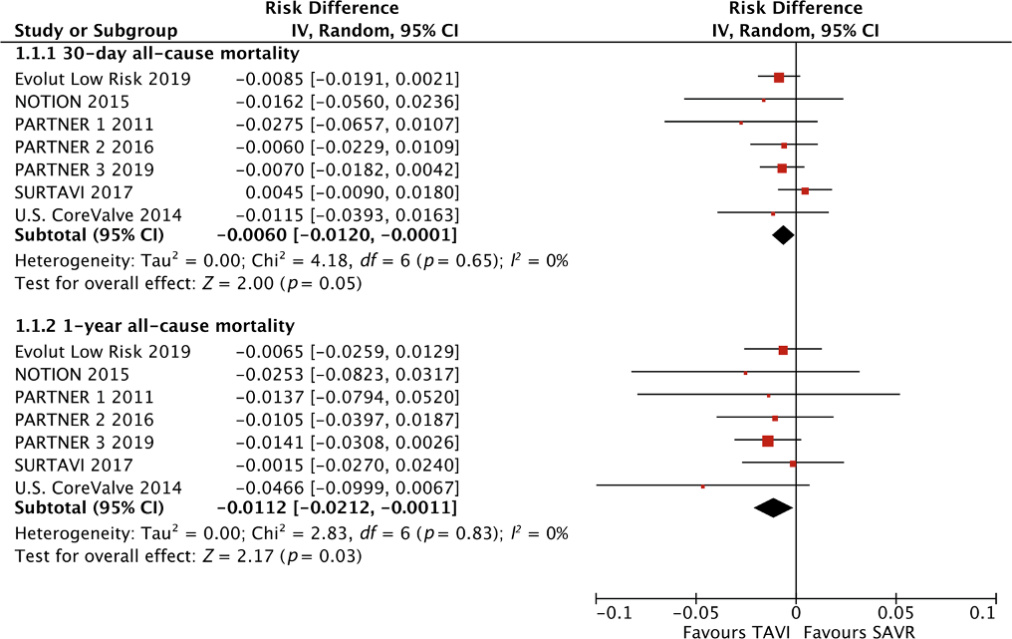
Forest plot of the principal analysis (as-treated population) of the primary end point: risk differences in 30-day and 1‑year all-cause mortality between transcatheter aortic valve implantation (*TAVI*) and surgical aortic valve replacement (*SAVR*). *CI* confidence interval, *IV* inverse variance, *NOTION* Nordic Aortic Valve Intervention, *PARTNER* Placement of Aortic Transcatheter Valves, *SURTAVI* Surgical Replacement and Transcatheter Aortic Valve Implantation

**Fig. 2:**
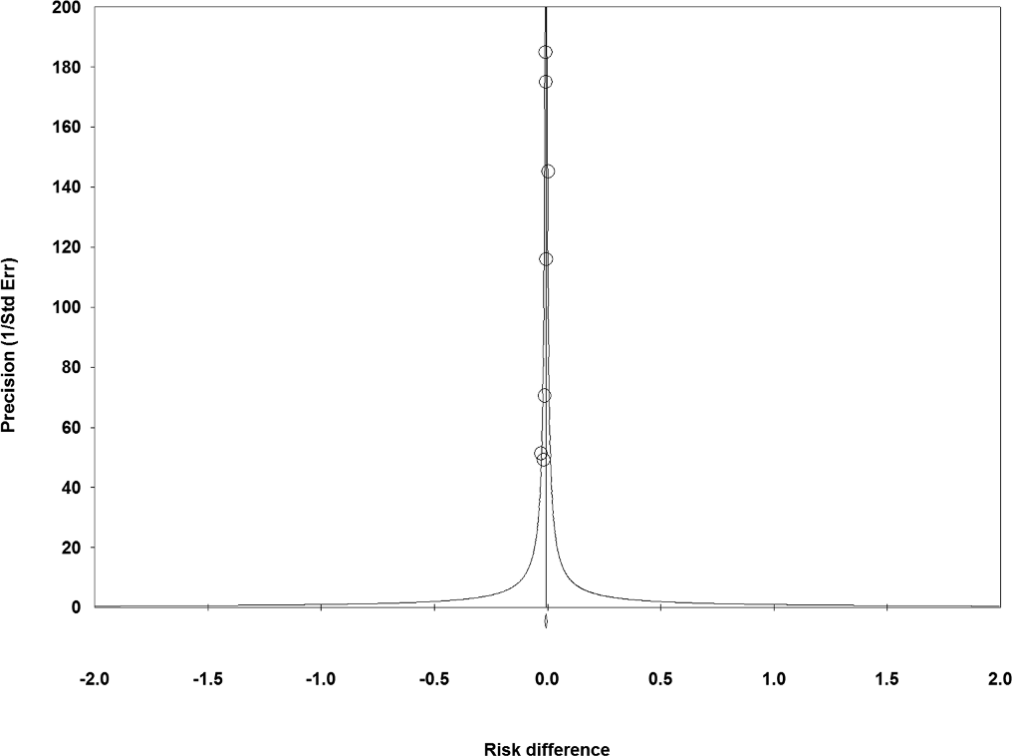
Funnel plot of the principal analysis (as-treated population) of the primary end point: precision by risk differences in 30-day all-cause mortality between transcatheter aortic valve implantation and surgical aortic valve replacement

**Fig. 3:**
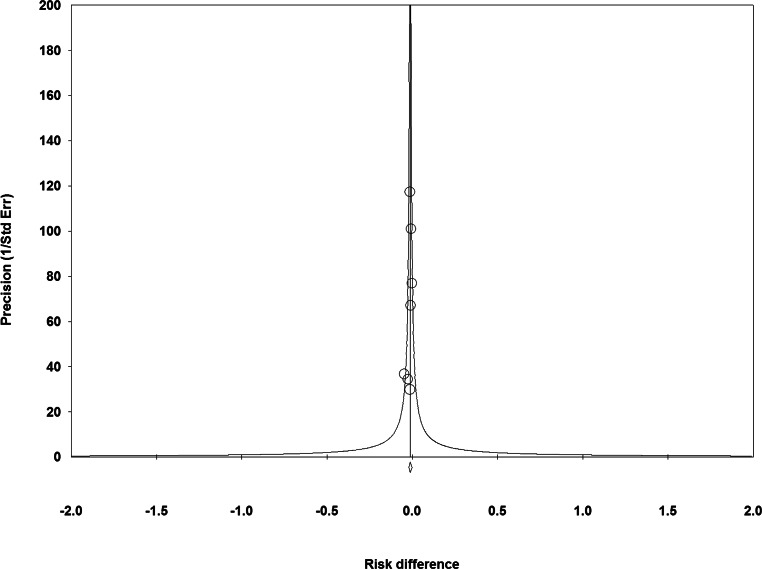
Funnel plot of the principal analysis (as-treated population) of the primary end point: precision by risk differences in 1‑year all-cause mortality between transcatheter aortic valve implantation and surgical aortic valve replacement

**Fig. 4:**
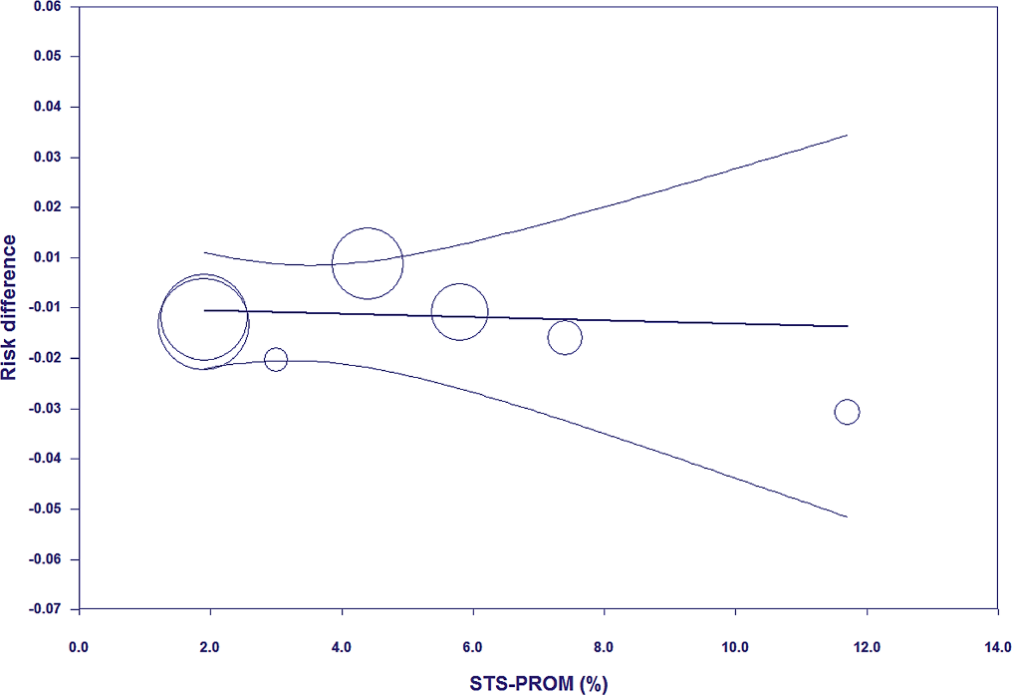
Meta-regression plot (meta-regression line with 95% confidence interval curves) of the principal analysis (as-treated population) of the primary end point: risk differences in 30-day all-cause mortality (between transcatheter aortic valve implantation and surgical aortic valve replacement) on Society of Thoracic Surgeons Predicted Risk of Mortality (*STS-PROM*) score

**Fig. 5:**
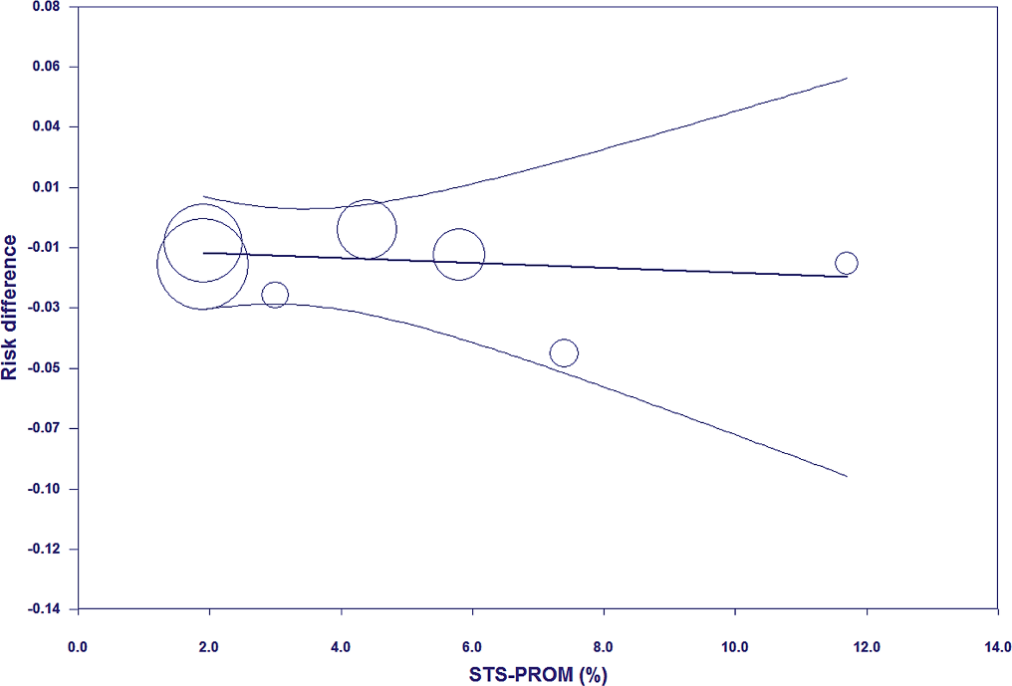
Meta-regression plot (meta-regression line with 95% confidence interval curves) of the principal analysis (as-treated population) of the primary end point: risk differences in 1‑year all-cause mortality (between transcatheter aortic valve implantation and surgical aortic valve replacement) on Society of Thoracic Surgeons Predicted Risk of Mortality (*STS-PROM*) score

None of the included RCTs showed significantly lower all-cause mortality after TAVI than after SAVR (Fig. 1). The principal analysis of the primary end point demonstrated significantly lower 30-day [RD −0.60%; 95% CI −1.20% to −0.01%; *p* = 0.05 (0.046, calculated using Comprehensive Meta-Analysis version 3); *I*^2^ 0%] and 1‑year all-cause mortality (RD −1.12%; 95% CI −2.12% to −0.11%; *p* = 0.03; *I*^2^ 0%) after TAVI than after SAVR (Fig. 1). No funnel plot asymmetry was detected for 30-day (*p* = 0.29; Fig. 2) and 1‑year mortality (*p* = 0.26; Fig. 3), which suggested no publication bias. Meta-regression analyses indicated that RDs of 30-day and 1‑year mortality (*p* = 0.73; Fig. 5) between TAVI and SAVR were not modulated by mean STS-PROM (*p* for 30-day/1-year mortality = 0.82/0.73; Figs. 4 and 5) and proportion of patients undergoing TF-TAVI (*p* for 30-day/1-year mortality = 0.73/0.50).

Results of the sensitivity analysis of the primary end point (all-cause mortality) are illustrated in Supplementary Fig. S1, and those of the principal and sensitivity analysis of the secondary end points (myocardial infarction, stroke, bleeding complications, AKI, vascular complications, and new permanent PMI) are diagramed in Supplementary Figs. S2–S10. Bleeding complications at 30 days and 1 year (Supplementary Figs. S6 and S7) and stage 2 or 3 AKI at 30 days (Supplementary Fig. S8) were significantly less frequent after TAVI than after SAVR, whereas major vascular complications (Supplementary Fig. S9) and new permanent PMI (Supplementary Fig. S10) at 30 days and 1 year were significantly more frequent after TAVI than after SAVR. There were no statistically significant differences in myocardial infarction (Supplementary Figs. S2 and S3) and stroke (Supplementary Figs. S4 and S5) at 30 days and 1 year between TAVI and SAVR.

## Discussion

The present study is the first meta-analysis (of RCTs) demonstrating that TAVI improves 30-day and 1‑year all-cause mortality compared with SAVR for AS patients. The absolute risk reduction was low, 0.60% for 30-day mortality and 1.12% for 1‑year mortality, but statistically significant. The present findings must be novel because none of the included RCTs showed significantly lower all-cause mortality after TAVI than after SAVR.

In the present principal analysis, data in the as-treated (not intention-to-treat) population were abstracted from each study and then combined because five of the seven RCTs principally analysed the as-treated population. To draw an unbiased estimate of the effect of the randomised treatment on the outcome, in general, the intention-to-treat analysis is recommended [13]. If some participants do not receive the randomised treatment, however, the intention-to-treat analysis may provide a biased estimate of the effect of the received treatment on the outcome. The as-treated analysis compares patients according to the received treatment rather than the randomised treatment, i.e. those who received the experimental treatment (whether or not they had been randomised to the experimental treatment) versus those who received the control treatment (whether or not they had been randomised to the control treatment) [13]. Thus, the as-treated analysis draws an unbiased estimate of the effect of the received treatment on the outcome. Clinicians or patients may be interested in whether the patient’s prognosis improves if the patient receives the experimental treatment (not if the patient is randomised to the experimental treatment) [13].

We extracted RDs of mortality from each study and then combined them in the present meta-analysis. Although simplicity for interpretation purposes is recognised to be a qualitative property, an RD would be agreed to be a simple measure and thus easily understood [14]. An RD advantage of, for example, 10% in the mortality rates of the experimental group relative to the control group is exactly equal to an RD disadvantage of 10% of the control group relative to the experimental group, which provides a symmetrical measure unaffected by labelling of study groups. In contradistinction to a risk or odds ratio estimate, an unbiased RD estimate is able to be gained from sample data based on the difference of two independent binomial variables [14].

Significantly lower 30-day and 1‑year all-cause mortality (Fig. 1) after TAVI than after SAVR (in the present principal analysis of the primary end point) could be explained by significantly less frequent bleeding complications at 30 days and 1 year (Supplementary Figs. S6 and S7) and stage 2 or 3 AKI (Supplementary Fig. S8) after TAVI than after SAVR (in the present principal analysis of the secondary end points). In the meta-analysis by Wang et al. [15] of 10 studies with a total of 3602 patients undergoing TAVI, bleeding complications were associated with a 323% increase in 30-day all-cause mortality [odds ratio (OR) 4.23; 95% CI 2.80–6.40; *p* < 0.0001], and major or life-threatening bleeding complications showed a 410% increase in 30-day all-cause mortality (OR 5.10; 95% CI 3.17–8.19; *p* < 0.0001). Furthermore, Liao et al. [16] demonstrated, in their meta-analysis of 35 studies with a total of 13,256 patients undergoing TAVI, that the aggravating severity of AKI was progressively associated with short-term all-cause mortality (univariate OR of 30-day mortality for stage 1, 3.41; for stage 2, 4.0; for stage 3, 11.02; univariate OR of 1‑year mortality for stage 1, 1.95; stage 2, 2.82; stage 3, 7.34). Even after controlling confounders, AKI was independently associated with a higher risk of both 30-day [multivariate hazard ratio (HR) 2.12; 95% CI 1.59–2.83] and ≥3-year all-cause mortality (multivariate HR 1.37; 95% CI 1.27–1.48) [16]. Faster and better recovery of left ventricular function [17] and less frequent pulmonary complications [18] after TAVI than after SAVR may also explain the present results. In patients with left ventricular systolic dysfunction, ejection fraction was reported to improve significantly (*p* < 0.05) better at 7 days after TAVI (32 ± 9% to 50 ± 17%) than after SAVR (30 ± 5% to 40 ± 9%) [17]. The total number of in-hospital pulmonary complications per patient was reported to be significantly (*p* = 0.04) lower after TF-TAVI (1.0 ± 0.67) than after SAVR (1.8 ± 0.79) [18]. However, significantly more frequent major vascular complications (Supplementary Fig. S9) and new permanent PMI (Supplementary Fig. S10) after TAVI than after SAVR (in the present principal analysis of the secondary end points) might potentially increase all-cause mortality. Vascular complications are strongly (approximately 3‑fold) associated with increased 30-day severe bleeding [19], which may affect 30-day survival after TAVI [15]. Several meta-analyses [20–22], however, indicated that a new permanent PMI was not associated with increased all-cause mortality during follow-up (up to 2 years) after TAVI. Although only the PARTNER 3 trial [6] with low-risk patients indicated a significantly lower incidence of stroke at 1 year (not at 30 days) after TAVI than after SAVR, the other RCTs demonstrated no significant difference in stroke at 30 days and 1 year between TAVI and SAVR, which brought about no significant difference in stroke in the present meta-analysis pooling all the RCTs (Supplementary Fig. S4).

Previous meta-analyses [4, 23–30] indicated no difference in 1‑year mortality between TAVI and SAVR. However, these meta-analyses [4, 23–30] (including neither the Evolut Low Risk trial [5] nor the PARTNER 3 trial [6]) were quite different from the present meta-analysis (including both the Evolut Low Risk trial [5] and the PARTNER 3 trial [6]). Longer (≥2-year)-term outcomes after TAVI versus SAVR in RCTs have still been inadequate. The longest follow-up durations were 1 year in two RCTs (Evolut Low Risk [5] and PARTNER 3 [6]), 2 years in two RCTs (PARTNER 2 [10] and SURTAVI [11]), and 5 years in three RCTs (NOTION [31], PARTNER 1 [32], and CoreValve U.S. Pivotal High Risk [33]). The present meta-analysis did not analyse ≥2-year mortality after TAVI versus SAVR. There were no statistically significant differences in all-cause mortality between TAVI and SAVR at 2 years (16.3% vs 17.9% [10], 12.6% vs 14.0% [11]) and 5 years (27.7% vs 27.7% [31], 74.01% vs 67.72% [32], 55.3% vs 55.4% [33]) in the as-treated population, despite significantly lower 30-day and 1‑year mortality after TAVI than after SAVR being demonstrated in the present study. This ‘catch-up’ phenomenon at ≥2 years may be owing to more frequent moderate/severe paravalvular aortic regurgitation [4, 34–37] and new-onset left bundle branch block [38], which are associated with higher ≥1-year all-cause [39, 40] and cardiac [41] mortality after TAVI than after SAVR. Although the 5‑year results of the Evolut Low Risk, PARTNER 2, PARTNER 3, and SURTAVI trials would be expected in the future, long-term mortality after TAVI might be similar to that after SAVR.

The present study had the following limitations. First, the RCTs included in the present meta-analysis were heterogeneous. Of the seven RCTs, three consisted of patients at low risk (STS-PROM <4%), three were composed of those at intermediate risk (STS-ROM 4–8%), and only one was made up of those at high risk (STS-ROM ≥8%). The proportion of patients undergoing TF-TAVI also ranged from 70.1 to 100% (Tab. 1). The meta-regression analyses, however, demonstrated that mean STS-PROM of patients and proportion of patients undergoing TF-TAVI did not modulate RDs of mortality between TAVI and SAVR. Furthermore, various TAVI valves were used in the RCTs and included CoreValve [5, 8, 11, 12], Evolut R [5, 11], Evolut PRO [5], SAPIEN [9], SAPIEN XT [10], and SAPIEN 3 [6], which may bias the present results. Second, although publication bias favouring TAVI may influence the present results, the funnel plot analysis did not indicate funnel plot asymmetry. Third, the cause of death was not addressed in the present meta-analysis because detailed patient-level data in all RCTs were unavailable. Individual patient data meta-analysis, the gold standard regarding data availability, would be required.

In conclusion, the best evidence from the present meta-analysis of all the currently available RCTs suggests that TAVI may reduce 30-day and 1‑year all-cause mortality compared with SAVR for AS patients. The present findings must be novel because none of the included RCTs showed significantly lower all-cause mortality after TAVI than after SAVR.

## Caption Electronic Supplementary Material

Supplementary Figs S1–S10

Supplementary Table S1

## References

[1] Kheiri B, Osman M, Abubakar H (2019). Transcatheter versus surgical aortic valve replacement in low-risk surgical patients: a meta-analysis of randomized clinical trials. Cardiovasc Revasc Med.

[2] Liu Z, Kidney E, Bem D (2018). Transcatheter aortic valve implantation for aortic stenosis in high surgical risk patients: a systematic review and meta-analysis. PLoS ONE.

[3] Wang Y, Zhou Y, Zhang L, Zhu J (2018). Midterm outcome of transcatheter versus surgical aortic valve replacement in low to intermediate risk patients: a meta-analysis of randomized controlled trials. J Cardiol.

[4] Burrage M, Moore P, Cole C (2017). Transcatheter aortic valve replacement is associated with comparable clinical outcomes to open aortic valve surgery but with a reduced length of in-patient hospital stay: a systematic review and meta-analysis of randomised trials. Heart Lung Circ.

[5] Popma JJ, Deeb GM, Yakubov SJ (2019). Evolut Low Risk Trial Investigators. Transcatheter aortic-valve replacement with a self-expanding valve in low-risk patients. N Engl J Med.

[6] Mack MJ, Leon MB, Thourani VH, PARTNER 3 Investigators (2019). Transcatheter aortic-valve replacement with a balloon-expandable valve in low-risk patients. N Engl J Med.

[7] Nielsen HH, Klaaborg KE, Nissen H (2012). A prospective, randomised trial of transapical transcatheter aortic valve implantation vs. surgical aortic valve replacement in operable elderly patients with aortic stenosis: the STACCATO trial. EuroIntervention.

[8] Thyregod HG, Steinbrüchel DA, Ihlemann N (2015). Transcatheter versus surgical aortic valve replacement in patients with severe aortic valve stenosis: 1-year results from the all-comers NOTION randomized clinical trial. J Am Coll Cardiol.

[9] Smith CR, Leon MB, Mack MJ, PARTNER Trial Investigators (2011). Transcatheter versus surgical aortic-valve replacement in high-risk patients. N Engl J Med.

[10] Leon MB, Smith CR, Mack MJ, PARTNER 2 Investigators (2016). Transcatheter or surgical aortic-valve replacement in intermediate-risk patients. N Engl J Med.

[11] Reardon MJ, Van Mieghem NM, Popma JJ, SURTAVI Investigators (2017). Surgical or transcatheter aortic-valve replacement in intermediate-risk patients. N Engl J Med.

[12] Adams DH, Popma JJ, Reardon MJ, U.S. CoreValve Clinical Investigators (2014). Transcatheter aortic-valve replacement with a self-expanding prosthesis. N Engl J Med.

[13] Shrier I, Steele RJ, Verhagen E, Herbert R, Riddell CA, Kaufman JS (2014). Beyond intention to treat: what is the right question?. Clin Trials.

[14] Walter SD (2000). Choice of effect measure for epidemiological data. J Clin Epidemiol.

[15] Wang J, Yu W, Jin Q (2017). Risk factors for post-TAVI bleeding according to the VARC-2 bleeding definition and effect of the bleeding on short-term mortality: a meta-analysis. Can J Cardiol.

[16] Liao YB, Deng XX, Meng Y (2017). Predictors and outcome of acute kidney injury after transcatheter aortic valve implantation: a systematic review and meta-analysis. EuroIntervention.

[17] Bauer F, Coutant V, Bernard M (2013). Patients with severe aortic stenosis and reduced ejection fraction: earlier recovery of left ventricular systolic function after transcatheter aortic valve implantation compared with surgical valve replacement. Echocardiography.

[18] Pettet JK, McGhee MN, McIlrath ST, Collins GL (2014). Comparison of pulmonary complications in patients undergoing transcatheter aortic valve implantation versus open aortic valve replacement. J Cardiothorac Vasc Anesth.

[19] Sun Y, Liu X, Chen Z (2017). Meta-analysis of predictors of early severe bleeding in patients who underwent transcatheter aortic valve implantation. Am J Cardiol.

[20] Escárcega RO, Magalhaes MA, Lipinski MJ (2014). Mortality in patients requiring pacemaker implantation following transcatheter aortic valve replacement: insights from a systematic review and meta-analysis. Int J Cardiol.

[21] Regueiro A, Abdul-Jawad Altisent O (2016). Impact of new-onset left bundle branch block and periprocedural permanent pacemaker implantation on clinical outcomes in patients undergoing transcatheter aortic valve replacement: a systematic review and meta-analysis. Circ Cardiovasc Interv.

[22] Mohananey D, Jobanputra Y, Kumar A (2017). Clinical and echocardiographic outcomes following permanent pacemaker implantation after transcatheter aortic valve replacement: meta-analysis and meta-regression. Circ Cardiovasc Interv.

[23] Ueshima D, Fovino LN, D’Amico G, Brener SJ, Esposito G, Tarantini G (2019). Transcatheter versus surgical aortic valve replacement in low- and intermediate-risk patients: an updated systematic review and meta-analysis. Cardiovasc Interv Ther.

[24] Arora S, Vaidya SR, Strassle PD (2018). Meta-analysis of transfemoral TAVR versus surgical aortic valve replacement. Catheter Cardiovasc Interv.

[25] Singh K, Carson K, Rashid MK (2018). Transcatheter aortic valve implantation in intermediate surgical risk patients with severe aortic stenosis: a systematic review and meta-analysis. Heart Lung Circ.

[26] Khan SU, Lone AN, Saleem MA, Kaluski E (2017). Transcatheter vs surgical aortic-valve replacement in low- to intermediate-surgical-risk candidates: a meta-analysis and systematic review. Clin Cardiol.

[27] Tam DY, Vo TX, Wijeysundera HC (2017). Transcatheter vs surgical aortic valve replacement for aortic stenosis in low-intermediate risk patients: a meta-analysis. Can J Cardiol.

[28] Garg A, Rao SV, Visveswaran G (2017). Transcatheter aortic valve replacement versus surgical valve replacement in low-intermediate surgical risk patients: a systematic review and meta-analysis. J Invasive Cardiol.

[29] Sardar P, Kundu A, Chatterjee S (2017). Transcatheter versus surgical aortic valve replacement in intermediate-risk patients: evidence from a meta-analysis. Catheter Cardiovasc Interv.

[30] Zhou Y, Wang Y, Wu Y, Zhu J (2017). Transcatheter versus surgical aortic valve replacement in low to intermediate risk patients: a meta-analysis of randomized and observational studies. Int J Cardiol.

[31] Thyregod HGH, Ihlemann N, Jørgensen TH (2019). Five-year clinical and echocardiographic outcomes from the nordic aortic valve intervention (NOTION) randomized clinical trial in lower surgical risk patients. Circulation.

[32] Mack MJ, Leon MB, Smith CR, PARTNER 1 trial investigators (2015). 5-year outcomes of transcatheter aortic valve replacement or surgical aortic valve replacement for high surgical risk patients with aortic stenosis (PARTNER 1): a randomised controlled trial. Lancet.

[33] Gleason TG, Reardon MJ, Popma JJ (2018). CoreValve U.S. pivotal high risk trial clinical investigators. 5-year outcomes of self-expanding transcatheter versus surgical aortic valve replacement in high-risk patients. J Am Coll Cardiol.

[34] Khan AR, Khan S, Riaz H (2016). Efficacy and safety of transcatheter aortic valve replacement in intermediate surgical risk patients: a systematic review and meta-analysis. Catheter Cardiovasc Interv.

[35] Indraratna P, Tian DH, Yan TD, Doyle MP, Cao C (2016). Transcatheter aortic valve implantation versus surgical aortic valve replacement: a meta-analysis of randomized controlled trials. Int J Cardiol.

[36] Gargiulo G, Sannino A, Capodanno D (2016). Transcatheter aortic valve implantation versus surgical aortic valve replacement: a systematic review and meta-analysis. Ann Intern Med.

[37] Arora S, Strassle PD, Ramm CJ (2017). Transcatheter versus surgical aortic valve replacement in patients with lower surgical risk scores: a systematic review and meta-analysis of early outcomes. Heart Lung Circ.

[38] Massoullié G, Bordachar P, Ellenbogen KA (2016). New-onset left bundle branch block induced by transcutaneous aortic valve implantation. Am J Cardiol.

[39] Takagi H, Umemoto T, ALICE (All-Literature Investigation of Cardiovascular Evidence) Group (2016). Impact of paravalvular aortic regurgitation after transcatheter aortic valve implantation on survival. Int J Cardiol.

[40] Athappan G, Patvardhan E, Tuzcu EM (2013). Incidence, predictors, and outcomes of aortic regurgitation after transcatheter aortic valve replacement: meta-analysis and systematic review of literature. J Am Coll Cardiol.

[41] Regueiro A, Abdul-Jawad Altisent O, Del Trigo M (2016). Impact of new-onset left bundle branch block and periprocedural permanent pacemaker implantation on clinical outcomes in patients undergoing transcatheter aortic valve replacement: a systematic review and meta-analysis. Circ Cardiovasc Interv.

