# Supplementary material for: Mortality after transcatheter versus surgical aortic valve replacement: an updated meta-analysis of randomised trials
Source: Neth Heart J. 2020 Mar 12;28(6):320–33. doi: 10.1007/s12471-020-01378-1 (PMC7270388; doi:10.1007/s12471-020-01378-1)
Supplement: Supplementary file 1 — Supplementary Figs S1–S10 [file 12471_2020_1378_MOESM1_ESM.pdf]

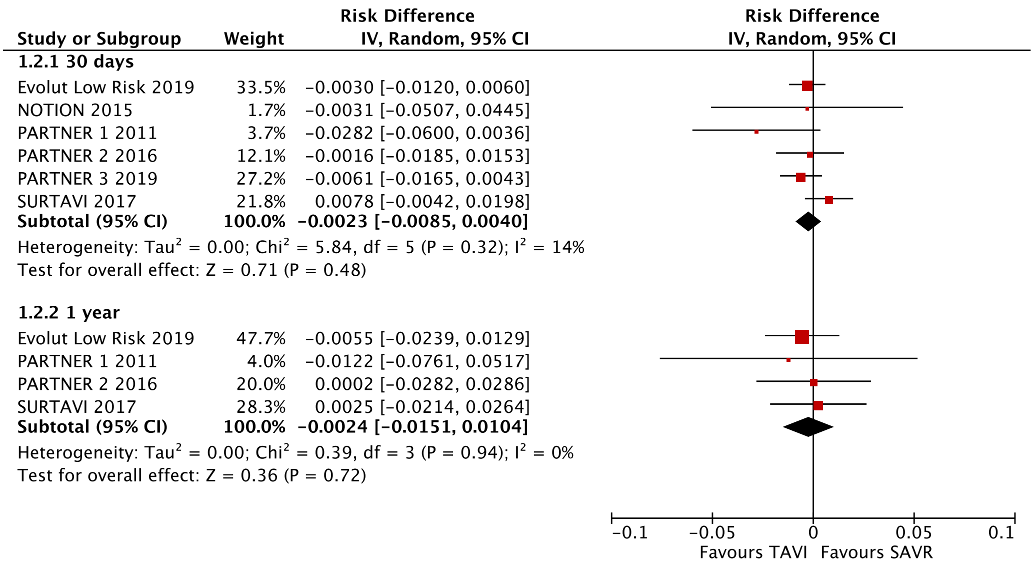

Supplementary Fig. S1 Forrest plot of the sensitivity analysis (intention-to-treat population) of the primary end-point: risk differences in 30-day and 1-year all-cause mortality between transcatheter aortic valve implantation (TAVI) and surgical aortic valve replacement (SAVR). *CI* confidence interval; *IV* inverse variance; *NOTION* Nordic Aortic Valve Intervention; *PARTNER* Placement of Aortic Transcatheter Valves; *SURTAVI* Surgical Replacement and Transcatheter Aortic Valve Implantation

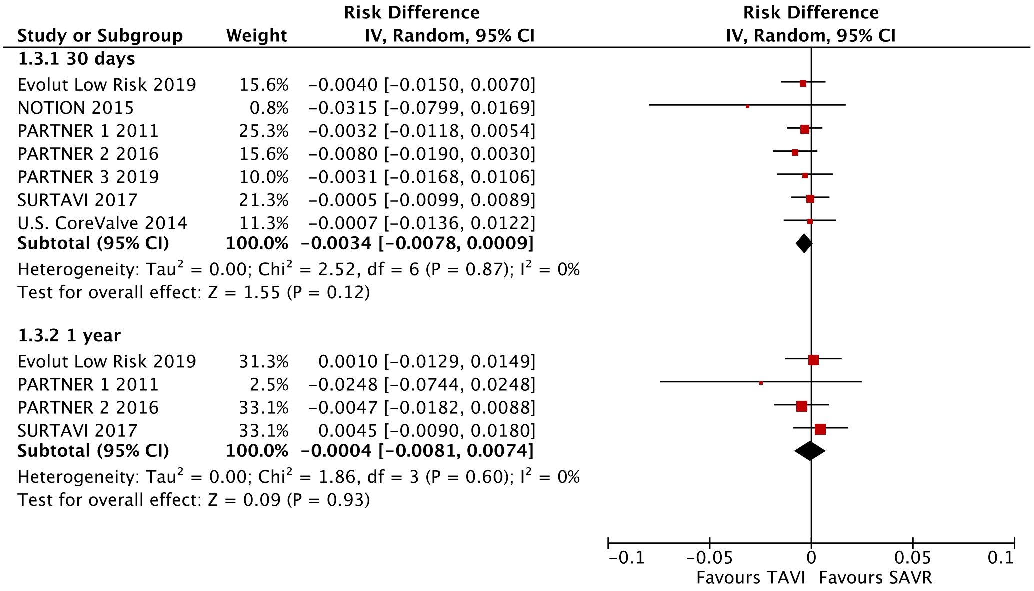

Supplementary Fig. S2 Forrest plot of the principal analysis (as-treated population) of the secondary end-point: risk differences in 30-day and 1-year myocardial infarction between transcatheter aortic valve implantation (TAVI) and surgical aortic valve replacement (SAVR). *CI* confidence interval; *IV* inverse variance; *NOTION* Nordic Aortic Valve Intervention; *PARTNER* Placement of Aortic Transcatheter Valves; *SURTAVI* Surgical Replacement and Transcatheter Aortic Valve Implantation

Study or Subgroup	Weight	Risk Difference IV, Random, 95% CI
**1.4.1 30 days**		
Evolut Low Risk 2019	26.1%	0.0025 [−0.0069, 0.0119]
PARTNER 1 2011	25.0%	−0.0057 [−0.0153, 0.0039]
PARTNER 2 2016	20.6%	−0.0067 [−0.0173, 0.0039]
SURTAVI 2017	28.4%	0.0020 [−0.0070, 0.0110]
**Subtotal (95% CI)**	**100.0%**	**−0.0016 [−0.0064, 0.0032]**
Heterogeneity: $Tau^2 = 0.00$; $Chi^2 = 2.93$, df = 3 (P = 0.40); $I^2 = 0\%$		
Test for overall effect: Z = 0.65 (P = 0.52)		
**1.4.2 1 year**		
Evolut Low Risk 2019	18.1%	0.0005 [−0.0140, 0.0150]
PARTNER 1 2011	41.3%	−0.0028 [−0.0124, 0.0068]
PARTNER 2 2016	19.7%	−0.0047 [−0.0186, 0.0092]
SURTAVI 2017	20.8%	0.0035 [−0.0100, 0.0170]
**Subtotal (95% CI)**	**100.0%**	**−0.0013 [−0.0074, 0.0049]**
Heterogeneity: $Tau^2 = 0.00$; $Chi^2 = 0.87$, df = 3 (P = 0.83); $I^2 = 0\%$		
Test for overall effect: Z = 0.40 (P = 0.69)		

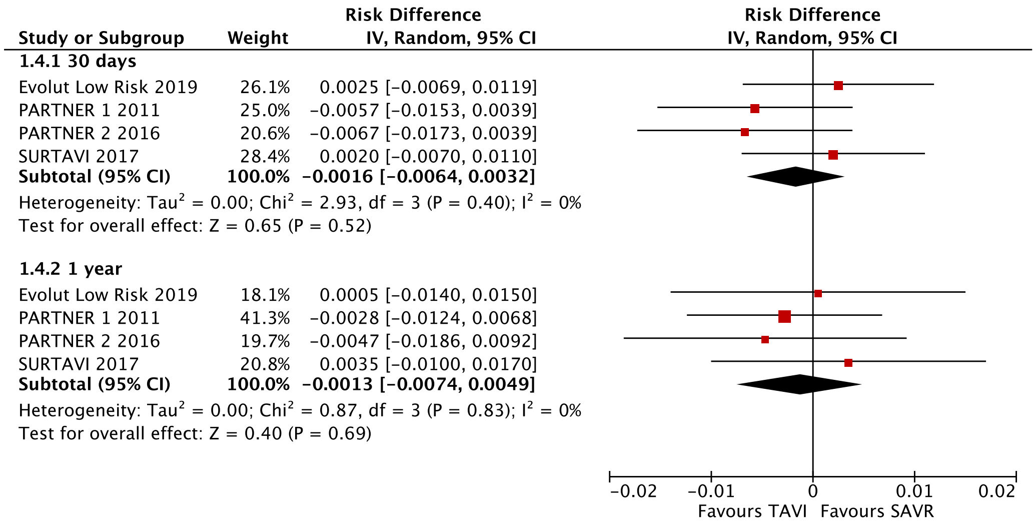

Supplementary Fig. S3 Forrest plot of the sensitivity analysis (intention-to-treat population) of the secondary end-point: risk differences in 30-day and 1-year myocardial infarction between transcatheter aortic valve implantation (TAVI) and surgical aortic valve replacement (SAVR). *CI* confidence interval; *IV* inverse variance; *PARTNER* Placement of Aortic Transcatheter Valves; *SURTAVI* Surgical Replacement and Transcatheter Aortic Valve Implantation

Study or Subgroup	Weight	Risk Difference IV, Random, 95% CI
**1.5.1 30 days**		
Evolut Low Risk 2019	25.5%	0.0000 [−0.0190, 0.0190]
NOTION 2015	7.8%	−0.0158 [−0.0505, 0.0189]
PARTNER 1 2011	11.8%	0.0210 [−0.0072, 0.0492]
PARTNER 2 2016	21.4%	−0.0050 [−0.0258, 0.0158]
PARTNER 3 2019	5.6%	−0.0147 [−0.0559, 0.0265]
SURTAVI 2017	19.3%	−0.0200 [−0.0420, 0.0020]
U.S. CoreValve 2014	8.7%	−0.0129 [−0.0458, 0.0200]
**Subtotal (95% CI)**	**100.0%**	**−0.0056 [−0.0154, 0.0041]**
Heterogeneity: $Tau^2 = 0.00$; $Chi^2 = 6.11$, df = 6 (P = 0.41); $I^2 = 2\%$		
Test for overall effect: Z = 1.13 (P = 0.26)		
**1.5.2 1 year**		
Evolut Low Risk 2019	18.9%	−0.0025 [−0.0241, 0.0191]
NOTION 2015	6.8%	−0.0166 [−0.0609, 0.0277]
PARTNER 1 2011	11.9%	0.0294 [−0.0016, 0.0604]
PARTNER 2 2016	16.6%	−0.0010 [−0.0251, 0.0231]
PARTNER 3 2019	22.0%	−0.0187 [−0.0373, −0.0001]
SURTAVI 2017	16.8%	−0.0150 [−0.0389, 0.0089]
U.S. CoreValve 2014	7.0%	−0.0330 [−0.0763, 0.0103]
**Subtotal (95% CI)**	**100.0%**	**−0.0072 [−0.0198, 0.0053]**
Heterogeneity: $Tau^2 = 0.00$; $Chi^2 = 9.20$, df = 6 (P = 0.16); $I^2 = 35\%$		
Test for overall effect: Z = 1.13 (P = 0.26)		

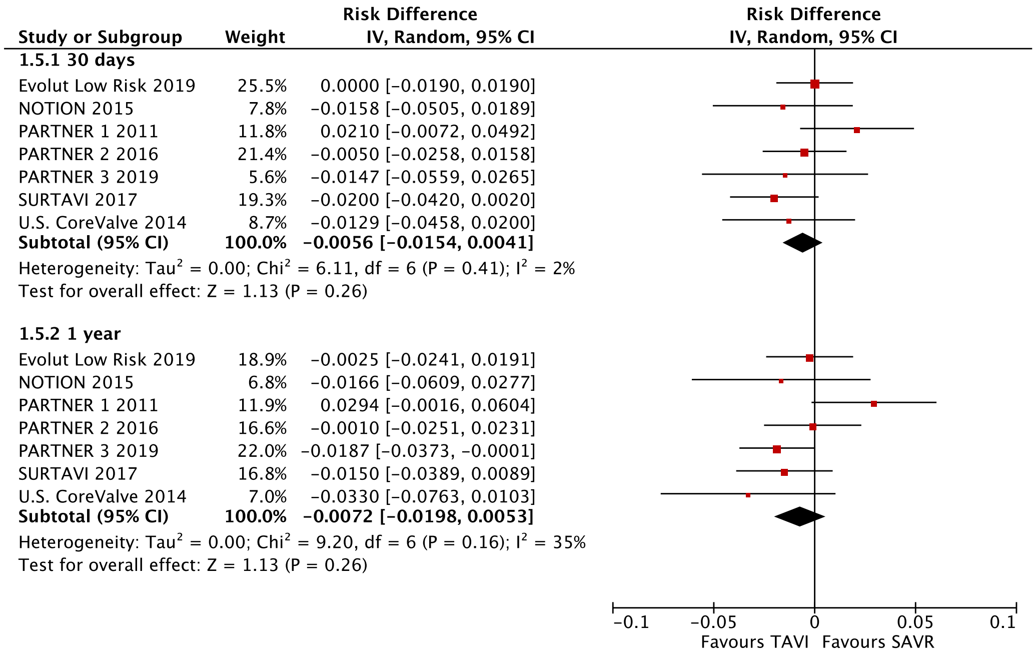

Supplementary Fig. S4 Forrest plot of the principal analysis (as-treated population) of the secondary end-point: risk differences in 30-day and 1-year stroke between transcatheter aortic valve implantation (TAVI) and surgical aortic valve replacement (SAVR). *CI* confidence interval; *IV* inverse variance; *NOTION* Nordic Aortic Valve Intervention; *PARTNER* Placement of Aortic Transcatheter Valves; *SURTAVI* Surgical Replacement and Transcatheter Aortic Valve Implantation

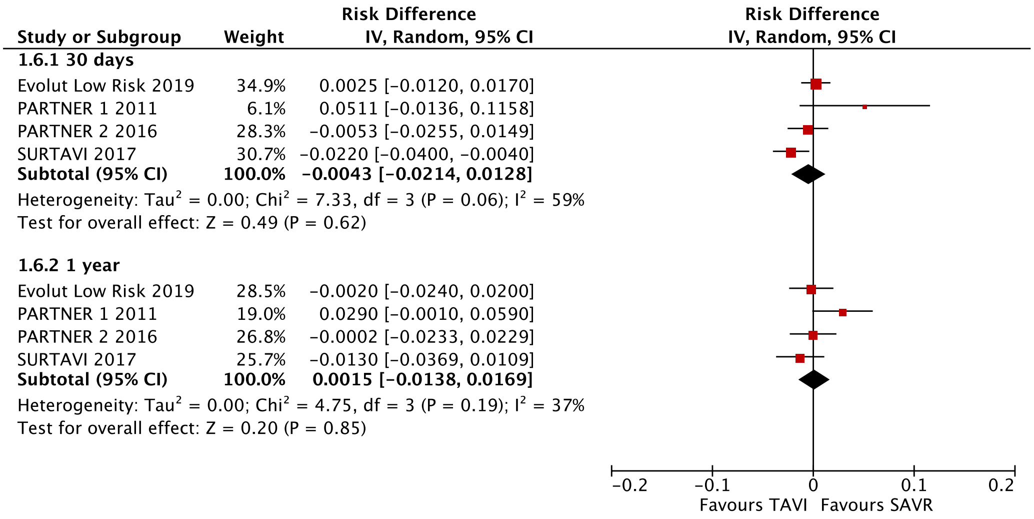

Supplementary Fig. S5 Forrest plot of the sensitivity analysis (intention-to-treat population) of the secondary end-point: risk differences in 30-day and 1-year stroke between transcatheter aortic valve implantation (TAVI) and surgical aortic valve replacement (SAVR). *CI* confidence interval; *IV* inverse variance; *NOTION* Nordic Aortic Valve Intervention; *PARTNER* Placement of Aortic Transcatheter Valves; *SURTAVI* Surgical Replacement and Transcatheter Aortic Valve Implantation

Study or Subgroup	Weight	Risk Difference IV, Random, 95% CI
**1.7.1 Major**		
PARTNER 1 2011	22.7%	−0.1251 [−0.1814, −0.0688]
PARTNER 3 2019	61.0%	−0.1082 [−0.1425, −0.0739]
U.S. CoreValve 2014	16.3%	−0.0651 [−0.1315, 0.0013]
**Subtotal (95% CI)**	**100.0%**	**−0.1050 [−0.1318, −0.0782]**
Heterogeneity: $Tau^2 = 0.00$; $Chi^2 = 1.91$, df = 2 (P = 0.38); $I^2 = 0\%$		
Test for overall effect: Z = 7.68 (P < 0.00001)		
**1.7.2 Life-threatening or disabling**		
Evolut Low Risk 2019	25.3%	−0.0520 [−0.0749, −0.0291]
PARTNER 2 2016	25.1%	−0.3636 [−0.4006, −0.3266]
PARTNER 3 2019	25.2%	−0.1068 [−0.1382, −0.0754]
U.S. CoreValve 2014	24.4%	−0.2142 [−0.2742, −0.1542]
**Subtotal (95% CI)**	**100.0%**	**−0.1835 [−0.3252, −0.0418]**
Heterogeneity: $Tau^2 = 0.02$; $Chi^2 = 206.42$, df = 3 (P < 0.00001); $I^2 = 99\%$		
Test for overall effect: Z = 2.54 (P = 0.01)		
**1.7.3 Major, life-threatening, or disabling**		
NOTION 2015	29.3%	−0.0963 [−0.1825, −0.0101]
PARTNER 3 2019	37.8%	−0.2082 [−0.2509, −0.1655]
U.S. CoreValve 2014	32.9%	−0.2793 [−0.3477, −0.2109]
**Subtotal (95% CI)**	**100.0%**	**−0.1988 [−0.2845, −0.1132]**
Heterogeneity: $Tau^2 = 0.00$; $Chi^2 = 10.62$, df = 2 (P = 0.005); $I^2 = 81\%$		
Test for overall effect: Z = 4.55 (P < 0.00001)		

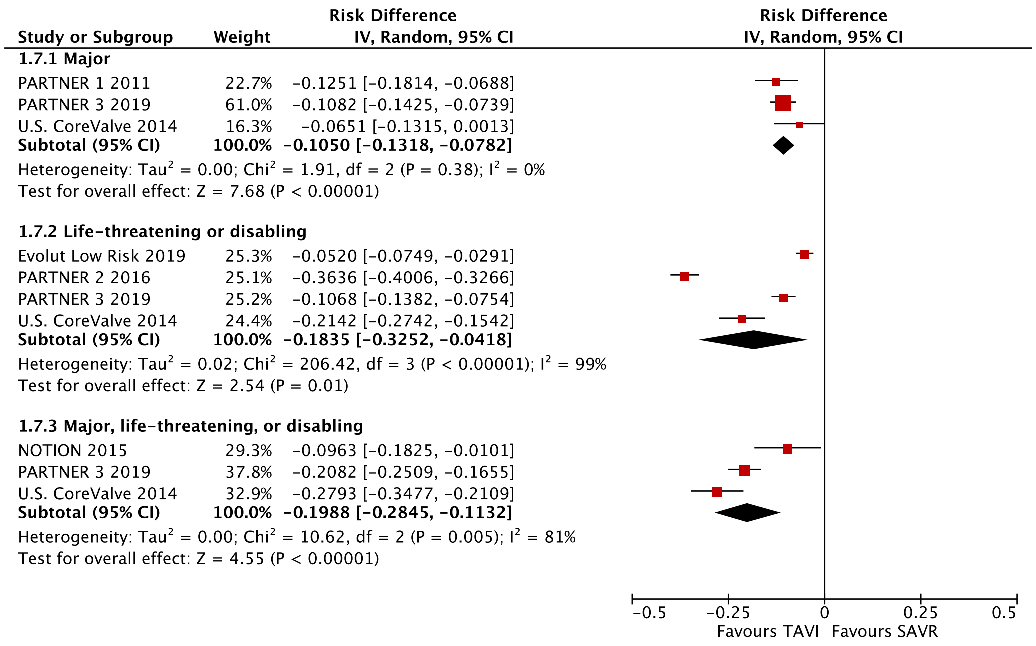

Supplementary Fig. S6 Forrest plot of the principal analysis (as-treated population) of the secondary end-point: risk differences in 30-day bleeding complications between transcatheter aortic valve implantation (TAVI) and surgical aortic valve replacement (SAVR). *CI* confidence interval; *IV* inverse variance; *NOTION* Nordic Aortic Valve Intervention; *PARTNER* Placement of Aortic Transcatheter Valves

Study or Subgroup	Weight	Risk Difference IV, Random, 95% CI
**1.8.1 Major**		
PARTNER 1 2011	54.8%	−0.1193 [−0.1801, −0.0585]
U.S. CoreValve 2014	45.2%	−0.0718 [−0.1390, −0.0046]
**Subtotal (95% CI)**	**100.0%**	**−0.0978 [−0.1442, −0.0515]**
Heterogeneity: $Tau^2 = 0.00$; $Chi^2 = 1.06$, df = 1 (P = 0.30); $I^2 = 5\%$		
Test for overall effect: Z = 4.14 (P < 0.0001)		
**1.8.2 Life-threatening or disabling**		
Evolut Low Risk 2019	33.5%	−0.0575 [−0.0840, −0.0310]
PARTNER 2 2016	33.2%	−0.3364 [−0.3752, −0.2976]
PARTNER 3 2019	33.3%	−0.0995 [−0.1334, −0.0656]
U.S. CoreValve 2014		Not estimable
**Subtotal (95% CI)**	**100.0%**	**−0.1640 [−0.3224, −0.0056]**
Heterogeneity: $Tau^2 = 0.02$; $Chi^2 = 140.55$, df = 2 (P < 0.00001); $I^2 = 99\%$		
Test for overall effect: Z = 2.03 (P = 0.04)		

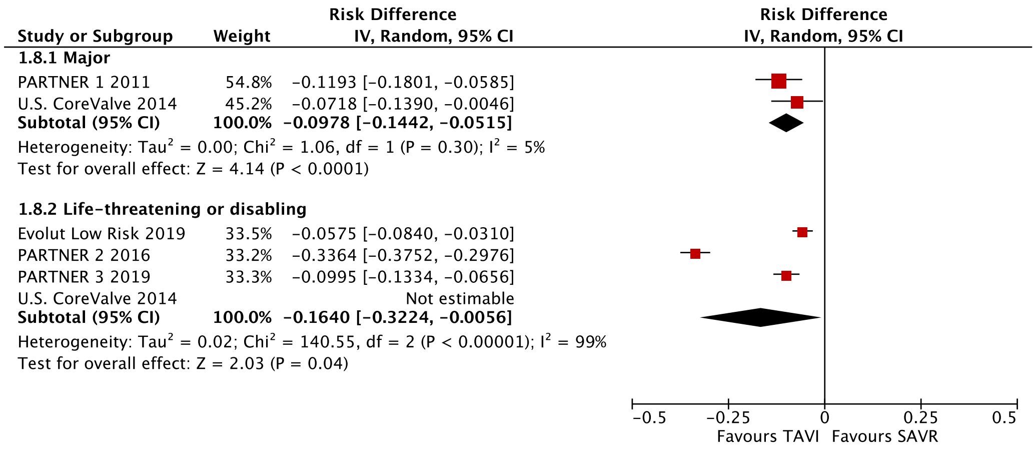

Supplementary Fig. S7 Forrest plot of the principal analysis (as-treated population) of the secondary end-point: risk differences in 1-year bleeding complications between transcatheter aortic valve implantation (TAVI) and surgical aortic valve replacement (SAVR). *CI* confidence interval; *IV* inverse variance; *NOTION* Nordic Aortic Valve Intervention; *PARTNER* Placement of Aortic Transcatheter Valves; *SURTAVI* Surgical Replacement and Transcatheter Aortic Valve Implantation

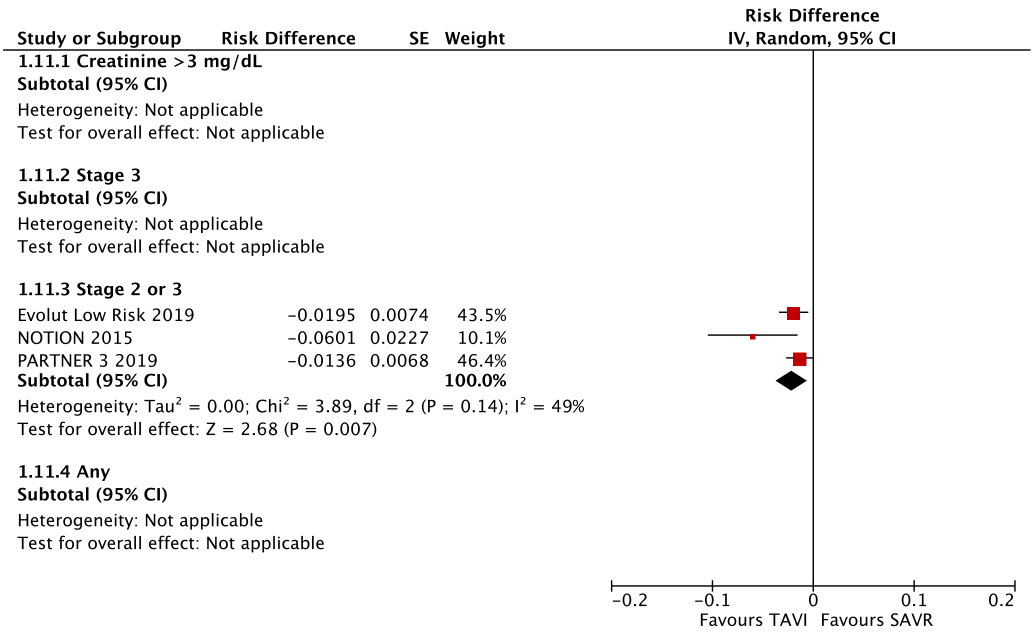

Supplementary Fig. S8 Forrest plot of the principal analysis (as-treated population) of the secondary end-point: risk differences in 30-day acute kidney injury between transcatheter aortic valve implantation (TAVI) and surgical aortic valve replacement (SAVR). *CI* confidence interval; *IV* inverse variance; *NOTION* Nordic Aortic Valve Intervention; *PARTNER* Placement of Aortic Transcatheter Valves; *SURTAVI* Surgical Replacement and Transcatheter Aortic Valve Implantation

Study or Subgroup	Risk Difference	SE	Weight
**1.15.1 30 days**			
Evolut Low Risk 2019	0.0055	0.0099	24.2%
NOTION 2015	0.0414	0.022	13.1%
PARTNER 1 2011	0.0753	0.0198	14.7%
PARTNER 2 2016	0.0265	0.0113	22.7%
PARTNER 3 2019	0.0068	0.0088	25.3%
**Subtotal (95% CI)**			**100.0%**
Heterogeneity: $Tau^2 = 0.00$; $Chi^2 = 13.28$, df = 4 (P = 0.010); $I^2 = 70\%$			
Test for overall effect: Z = 2.43 (P = 0.01)			
**1.15.2 1 year**			
Evolut Low Risk 2019	0.003	0.0102	27.7%
PARTNER 1 2011	0.0753	0.0198	17.5%
PARTNER 2 2016	0.0273	0.0116	26.1%
PARTNER 3 2019	0.0128	0.0094	28.6%
**Subtotal (95% CI)**			**100.0%**
Heterogeneity: $Tau^2 = 0.00$; $Chi^2 = 11.48$, df = 3 (P = 0.009); $I^2 = 74\%$			
Test for overall effect: Z = 2.12 (P = 0.03)			

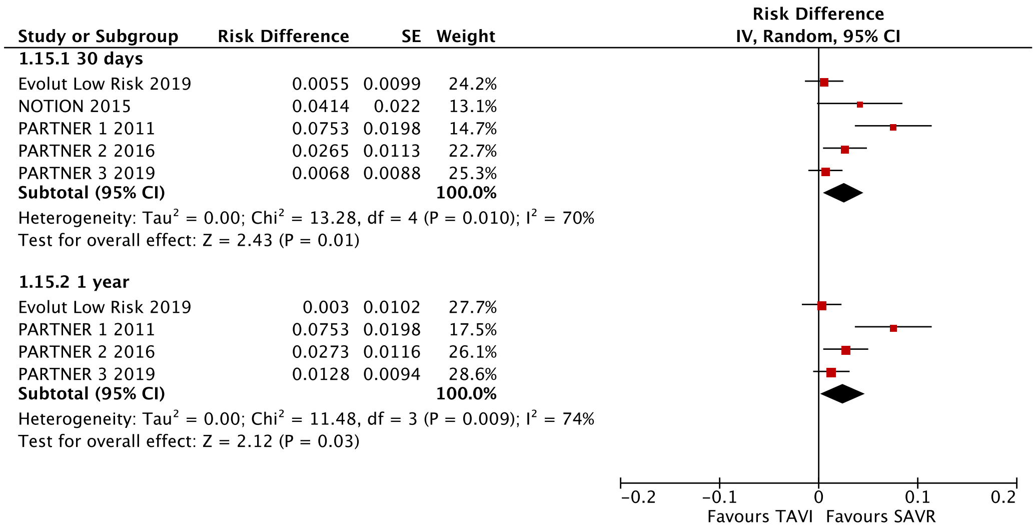

Supplementary Fig. S9 Forrest plot of the principal analysis (as-treated population) of the secondary end-point: risk differences in 30-day and 1-year major vascular complications between transcatheter aortic valve implantation (TAVI) and surgical aortic valve replacement (SAVR). *CI* confidence interval; *IV* inverse variance; *NOTION* Nordic Aortic Valve Intervention; *PARTNER* Placement of Aortic Transcatheter Valves; *SURTAVI* Surgical Replacement and Transcatheter Aortic Valve Implantation

Study or Subgroup	Risk Difference	SE	Weight
**1.17.1 30 days**			
Evolut Low Risk 2019	0.1135	0.0171	17.3%
NOTION 2015	0.309	0.0406	13.7%
PARTNER 1 2011	-0.0011	0.0161	17.4%
PARTNER 2 2016	0.0135	0.0122	17.8%
PARTNER 3 2019	0.0249	0.0143	17.6%
U.S. CoreValve 2014	0.1248	0.0242	16.3%
**Subtotal (95% CI)**			**100.0%**
Heterogeneity: $Tau^2 = 0.00$; $Chi^2 = 85.96$, df = 5 (P < 0.00001); $I^2 = 94\%$			
Test for overall effect: Z = 2.97 (P = 0.003)			
**1.17.2 1 year**			
Evolut Low Risk 2019	0.127	0.0179	17.3%
NOTION 2015	0.3368	0.0422	14.0%
PARTNER 1 2011	0.0012	0.0173	17.3%
PARTNER 2 2016	0.0085	0.0133	17.6%
PARTNER 3 2019	0.0197	0.0157	17.5%
U.S. CoreValve 2014	0.1115	0.0265	16.3%
**Subtotal (95% CI)**			**100.0%**
Heterogeneity: $Tau^2 = 0.01$; $Chi^2 = 91.44$, df = 5 (P < 0.00001); $I^2 = 95\%$			
Test for overall effect: Z = 2.78 (P = 0.005)			

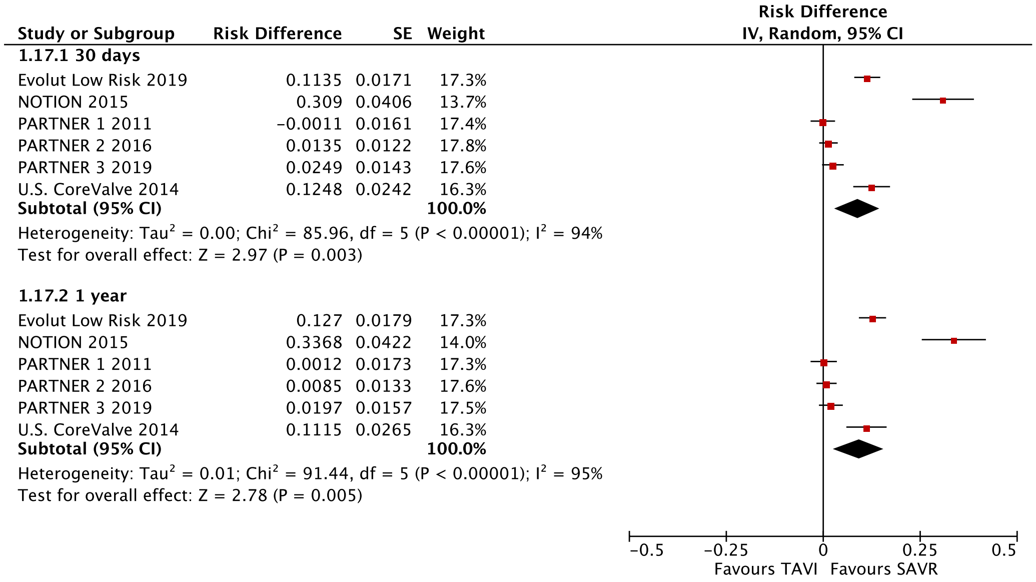

Supplementary Fig. S10 Forrest plot of the principal analysis (as-treated population) of the secondary end-point: risk differences in 30-day and 1-year new permanent pacemaker implantation between transcatheter aortic valve implantation (TAVI) and surgical aortic valve replacement (SAVR). *CI* confidence interval; *IV* inverse variance; *NOTION* Nordic Aortic Valve Intervention; *PARTNER* Placement of Aortic Transcatheter Valves
