# Supplementary material for: Mortality after transcatheter versus surgical aortic valve replacement: an updated meta-analysis of randomised trials
Source: Neth Heart J. 2020 Mar 12;28(6):320–33. doi: 10.1007/s12471-020-01378-1 (PMC7270388; doi:10.1007/s12471-020-01378-1)
Supplement: Supplementary file 2 — Supplementary Table S1 [file 12471_2020_1378_MOESM2_ESM.pdf]

Supplementary Table S1 Secondary end points (myocardial infarction, stroke, bleeding complications, acute kidney injury, vascular complications, and new permanent pacemaker implantation)

Study	Myocardial infarction															
	Principal analysis (as-treated population)								Sensitivity analysis (intention-to-treat population)							
	30 days				1 year				30 days				1 year			
	Number		Percentage		Number		Percentage		Number		Percentage		Number		Percentage	
	TAVI	SAVR	TAVI	SAVR	TAVI	SAVR	TAVI	SAVR	TAVI	SAVR	TAVI	SAVR	TAVI	SAVR	TAVI	SAVR
Evolut Low Risk 2019[5]	7	9	1.0	1.3	28	24	3.9	3.5	7	4	1.0	0.5	12	12	1.6	1.6
NOTION 2015[8]	4	8	2.8	6.0	5	8	3.5	6.0	Unavailable		Unavailable		Unavailable		Unavailable	
PARTNER 1 2011[9]	0	1	0.0	0.3	1	1	0.3	0.3	0	2	0.0	0.6	1	2	0.3	0.6
PARTNER 2 2016[10]	11	18	1.1	1.9	23	28	2.3	3.0	12	19	1.2	1.9	24	29	2.4	2.8
PARTNER 3 2019[6]	5	6	1.0	1.3	6	10	1.2	2.2	Unavailable		Unavailable		Unavailable		Unavailable	
SURTAVI 2017[11]	8	8	0.9	1.0	17	13	2.0	1.6	8	6	0.9	0.7	18	15	2.0	1.7
U.S. CoreValve 2014[12]	3	3	0.8	0.8	7	5	1.8	1.4	Unavailable		Unavailable		Unavailable		Unavailable	

NOTION Nordic Aortic Valve Intervention; *PARTNER* Placement of Aortic Transcatheter Valves; *SAVR* surgical aortic valve replacement; *SURTAVI* Surgical Replacement and Transcatheter Aortic Valve Implantation; *TAVI* transcatheter aortic valve implantation

Supplementary Table S1 *Continued*

Study	Stroke															
	Principal analysis (as-treated population)								Sensitivity analysis (intention-to-treat population)							
	30 days				1 year				30 days				1 year			
	Number		Percentage		Number		Percentage		Number		Percentage		Number		Percentage	
	TAVI	SAVR	TAVI	SAVR	TAVI	SAVR	TAVI	SAVR	TAVI	SAVR	TAVI	SAVR	TAVI	SAVR	TAVI	SAVR
Evolut Low Risk 2019[5]	25	23	3.4	3.4	30	29	4.1	4.3	15	14	2.0	1.9	29	31	4.0	4.2
NOTION 2015[8]	2	4	1.4	3.0	4	6	2.8	4.5	Unavailable		Unavailable		Unavailable		Unavailable	
PARTNER 1 2011[9]	16	8	4.7	2.6	20	9	5.8	2.9	16	8	4.6	2.3	20	10	5.7	2.8
PARTNER 2 2016[10]	55	57	5.5	6.0	78	75	7.8	7.9	55	61	5.4	6.0	78	79	7.7	7.7
PARTNER 3 2019[6]	3	11	0.6	2.4	6	14	1.2	3.1	Unavailable		Unavailable		Unavailable		Unavailable	
SURTAVI 2017[11]	29	45	3.4	5.7	47	55	5.4	6.9	23	42	2.6	4.8	48	59	5.5	6.8
U.S. CoreValve 2014[12]	19	22	4.9	6.2	33	42	8.5	11.8	Unavailable		Unavailable		Unavailable		Unavailable	

Supplementary Table S1 *Continued*

Study	Bleeding complications																
		Principal analysis (as-treated population)								Sensitivity analysis (intention-to-treat population)							
		30 days				1 year				30 days				1 year			
		Number		Percentage		Number		Percentage		Number		Percentage		Number		Percentage	
		TAVI	SAVR	TAVI	SAVR	TAVI	SAVR	TAVI	SAVR	TAVI	SAVR	TAVI	SAVR	TAVI	SAVR	TAVI	SAVR
Evolut Low Risk 2019[5]	Life-threatening or disabling	17	51	2.3	7.5	23	60	3.2	8.8	Unavailable		Unavailable		Unavailable		Unavailable	
NOTION 2015[8]	Major, life-threatening, or disabling	16	28	11.3	20.9	Unavailable		Unavailable		Unavailable		Unavailable		Unavailable		Unavailable	
PARTNER 1 2011[9]	Major	35	71	10.2	22.7	48	81	14.0	25.9	32	67	9.2	19.1	49	85	14.1	24.2
PARTNER 2 2016[10]	Life-threatening or disabling	104	442	10.5	46.8	150	460	15.1	48.7	105	442	10.4	43.3	151	460	14.9	45.1
PARTNER 3 2019[6]	Major	13	61	2.6	13.4	26	64	5.2	14.1	Unavailable		Unavailable		Unavailable		Unavailable	
	Life-threatening or disabling	6	54	1.2	11.9	14	58	2.8	12.8	Unavailable		Unavailable		Unavailable		Unavailable	
	Major, life-threatening, or disabling	18	111	3.6	24.4	38	117	7.7	25.8	Unavailable		Unavailable		Unavailable		Unavailable	
SURTAVI 2017[11]	Unavailable	Unavailable		Unavailable		Unavailable		Unavailable		Unavailable		Unavailable		Unavailable		Unavailable	
U.S. CoreValve 2014[12]	Major	109	123	27.9	34.5	114	130	29.2	36.4	Unavailable		Unavailable		Unavailable		Unavailable	
	Life-threatening or disabling	53	125	13.6	35.0	64	136	16.4	38.1	Unavailable		Unavailable		Unavailable		Unavailable	
	Major, life-threatening, or disabling	162	248	41.5	69.5	178	266	45.6	74.5	Unavailable		Unavailable		Unavailable		Unavailable	

Supplementary Table S1 *Continued*

Study	Acute kidney injury																
		Principal analysis (as-treated population)								Sensitivity analysis (intention-to-treat population)							
		30 days				1 year				30 days				1 year			
		Number		Percentage		Number		Percentage		Number		Percentage		Number		Percentage	
		TAVI	SAVR	TAVI	SAVR	TAVI	SAVR	TAVI	SAVR	TAVI	SAVR	TAVI	SAVR	TAVI	SAVR	TAVI	SAVR
Evolut Low Risk 2019[5]	Stage 2 or 3	7	19	1.0	2.8	7	19	1.0	2.8	Unavailable		Unavailable		Unavailable		Unavailable	
NOTION 2015[8]	Stage 2 or 3	1	9	0.7	6.7	Unavailable		Unavailable		Unavailable		Unavailable		Unavailable		Unavailable	
PARTNER 1 2011[9]	Creatinine >3 mg/dL	3	3	0.9	1.0	10	5	2.9	1.6	4	4	1.1	1.1	12	8	3.4	2.3
PARTNER 2 2016[10]	Stage 3	12	31	1.2	3.3	31	48	3.1	5.1	13	31	1.3	3.0	32	48	3.2	4.7
PARTNER 3 2019[6]	Stage 3	2	3	0.4	0.7	Unavailable		Unavailable		Unavailable		Unavailable		Unavailable		Unavailable	
	Stage 2 or 3	2	8	0.4	1.8	Unavailable		Unavailable		Unavailable		Unavailable		Unavailable		Unavailable	
SURTAVI 2017[11]	Unavailable	Unavailable		Unavailable		Unavailable		Unavailable		Unavailable		Unavailable		Unavailable		Unavailable	
U.S. CoreValve 2014[12]	Any	23	54	5.9	15.1	23	54	5.9	15.1	Unavailable		Unavailable		Unavailable		Unavailable	

Supplementary Table S1 *Continued*

Study	Major Vascular complications															
	Principal analysis (as-treated population)								Sensitivity analysis (intention-to-treat population)							
	30 days				1 year				30 days				1 year			
	Number		Percentage		Number		Percentage		Number		Percentage		Number		Percentage	
	TAVI	SAVR	TAVI	SAVR	TAVI	SAVR	TAVI	SAVR	TAVI	SAVR	TAVI	SAVR	TAVI	SAVR	TAVI	SAVR
Evolut Low Risk 2019[5]	28	22	3.9	3.2	28	24	3.9	3.5	Unavailable		Unavailable		Unavailable		Unavailable	
NOTION 2015[8]	8	2	5.6	1.5	Unavailable		Unavailable		Unavailable		Unavailable		Unavailable		Unavailable	
PARTNER 1 2011[9]	38	11	11.0	3.5	38	11	11.0	3.5	38	11	10.9	3.1	39	12	11.2	3.4
PARTNER 2 2016[10]	80	51	8.0	5.4	84	54	8.5	5.7	80	51	7.9	5.0	84	54	8.3	5.3
PARTNER 3 2019[6]	11	7	2.2	1.5	14	7	2.8	1.5	Unavailable		Unavailable		Unavailable		Unavailable	
SURTAVI 2017[11]	Unavailable		Unavailable		Unavailable		Unavailable		Unavailable		Unavailable		Unavailable		Unavailable	
U.S. CoreValve 2014[12]	Unavailable		Unavailable		Unavailable		Unavailable		Unavailable		Unavailable		Unavailable		Unavailable	
